# Antimicrobial Effectiveness of an Usnic-Acid-Containing Self-Decontaminating Coating on Underground Metro Surfaces in Athens

**DOI:** 10.3390/microorganisms10112233

**Published:** 2022-11-11

**Authors:** Helena C. Maltezou, Elina Horefti, Nikolaos Papamichalopoulos, Maria Tseroni, Anastasios Ioannidis, Emmanouil Angelakis, Stylianos Chatzipanagiotou

**Affiliations:** 1 Directorate of Research, Studies and Documentation, National Public Health Organization, 3–5 Agrafon Street, 15123 Athens, Greece; 2 Hellenic Pasteur Institute, 127 Vas. Sophias Ave, 11521 Athens, Greece; 3 Department of Medical Biopathology, Aeginition Hospital, Medical School, National and Kapodistrian University of Athens, 72–74 Vas. Sophias Ave, 11528 Athens, Greece; 4 Directorate for Epidemiological Surveillance of Infectious Diseases, National Public Health Organization, 3–5 Agrafon Street, 15123 Athens, Greece; 5 Department of Nursing, Faculty of Health Sciences, University of Peloponnese, Sehi Area, 22100 Tripoli, Greece

**Keywords:** SARS-CoV-2, bacteria, fungi, environmental contamination, usnic acid, underground metro

## Abstract

(1) Background: Surfaces have been implicated in the transmission of infections. We aimed to assess how effective an usnic-acid-containing self-decontaminating coating was on the surfaces of the Athens underground metro. (2) Methods: Two samples were collected from each of 60 surfaces of a station and a wagon before the application of the coating and 9 and 20 days after, and they were tested for bacteria, fungi, and SARS-CoV-2 using conventional microbiological and molecular methods. Bacteria and fungi growth were expressed in colony forming units (CFUs)/10^2^cm^2^. (3) Results: Before the application of the coating, 50% of the samples tested positive for the targeted microbes: 91.7% for bacteria, 18.3% for fungi, and 8.3% for SARS-CoV-2. After nine days, 3.3% of the samples tested positive for bacteria and 6.6% after 20 days. The average amount of bacteria before the coating was applied was 8.5 CFU/10^2^cm^2^ compared to 0 and 0 CFU/10^2^cm^2^ after application (100% and 95% reduction); all samples collected after the application were negative for SARS-CoV-2 and fungi (100% reduction). (4) Conclusion: An usnic-acid-containing self-decontaminating coating was highly effective in eliminating bacterial, fungal, and SARS-CoV-2 contamination of surfaces in the underground metro.

## 1. Introduction

Underground metros are important urban indoor environments used by millions of people on a daily basis. Large numbers of people often coexist under crowded conditions for a significant time period, which may facilitate the transmission of infections. Most environmental investigations in underground metros focus on airborne microbiomes [1,2], while several studies have shown the association between underground use and transmission of acute respiratory illnesses, including pandemic influenza [3,4,5,6].

Contamination of common public surfaces has been implicated in transmission of infections [7]. In recent years, the surface microbial load of several underground metros has been studied as a means to understand microbe–host interactions [8,9,10,11,12,13]. It has been shown that during an underground trip, 99% of passengers are in contact with surfaces, mostly with their hands, which, in turn, significantly expands their microbial diversity, similar to other passengers and to underground surfaces [12]. Self-contact and contact with personal items (e.g., cellular phones) is also common [12]. Notably, although routine cleaning procedures remove most bacteria, on many surfaces, bacterial composition re-establishes within minutes or several hours [12]. Moreover, microbial load diversity significantly correlates with passenger traffic [10]. A metagenome sequencing study on palm samples from passengers of various lines of the Hong Kong metro system showed that the detected bacteria were largely derived from human and skin commensals, following mass surface exposure and recolonization [13]. Lastly, although respiratory droplets are the prevalent route of SARS-CoV-2 transmission, the virus is readily detected on surfaces, where it can remain viable and infectious for up to four days [14]. Nevertheless, almost all studies on SARS-CoV-2 environmental contamination concern healthcare settings [15,16]. To our knowledge, published data on SARS-CoV-2 contamination in underground metros are scarce [17]. A model that simulated various scenarios of potential exposure to SARS-CoV-2 on a subway wagon predicted that SARS-CoV-2 transmission through touching contaminated surfaces is possible, second only to close exposure (<2 m) to an infectious passenger [18]. Therefore, the development and evaluation in real life of long-term strategies to eliminate the contamination of surfaces in underground metros are imperative [7].

Usnic acid, a dibenzofuran, was originally isolated from lichens. Usnic acid and its derivatives possess good antibacterial activity against a wide range of Gram-positive and Gram-negative bacteria, including multidrug-resistant (MDR) bacteria, such as *Staphylococcus aureus*, *S. epidermidis*, *Enterococcus faecalis*, and, to a lesser extent, against *Pseudomonas aeruginosa*, *Escherichia coli*, and meticillin-resistant *S. aureus* [19,20,21,22,23,24]. Moreover, usnic acid and its derivatives have good antifungal activity against *Candida spp and Aspergillus* spp. [22,25,26]. In several cases, the antibacterial and antifungal activity of usnic acid and its derivatives is comparable or surpasses the antibacterial and antifungal activity of many reference drugs [22]. The antibacterial and antifungal activity of usnic acid is conferred through disruption of cell membranes, but mainly through biofilm inhibition and adhesion prevention [20,23,26]. Usnic acid and its derivatives also demonstrate activity against influenza virus through suppression of virus replication [27,28].

Usnic acid has been integrated into a polymer, which, following procession, transforms to a crystal layer forming a protective, self-decontamination semi-liquid coating named Natural Protective Shield 360° (NPS 360°; hereafter referred to as coating). The coating confers antimicrobial protection, rendering its application a decontamination strategy for highly contaminated environments [29]. The coating is not classified as hazardous in accordance with Regulation No 1272/2008 of the European Parliament [29].

The aim of the current study was to assess the effectiveness of the usnic-acid-based self-decontaminating coating NPS 360° in reducing bacterial, fungal, and SARS-CoV-2 surface contamination in the Athens underground metro using real-life data.

## 2. Materials and Methods

### 2.1. Setting

The Athens underground metro, the most important transportation mode in Athens metropolitan area, extends over 39 km and includes 36 stations, through which 968,000 passengers are moved on a daily basis [30]. Cleaning and decontamination of stations and wagons are performed once daily after the last itinerary. Floor-cleaning machines and 4.5% bleach-based products are used in stations while a 1% ClO_2_-containing product is applied in wagons.

### 2.2. Collection of Environmental Samples

Environmental investigation was conducted in three sampling phases: sampling phase 1 (1 h before the application of the coating); sampling phase 2 (9 days after the application of the coating); and sampling phase 3 (20 days after the application of the coating). In particular, on 14 May 2022 (sampling phase 1) one trained professional collected environmental samples from Sepolia Station while on duty and from Wagon 69M317 at Wagons Terminal after the last itinerary but before routine cleaning and decontamination. Sixty heavily touched surfaces from the station and the wagon were selected for sampling. Thus, 2 samples were collected from each of the 60 preselected sampling surfaces at the same time (120 samples in total). Sampling surfaces included metallic objects (e.g., wagon poles, station seats), non-porous plastic objects (e.g., elevator buttons), fabric-covered objects (e.g., wagon seats), and porous rubber objects (e.g., escalator handrails and wagon handles). Samples were collected using sterile swabs in 2 mL of viral nucleic acid sample preservation fluid. The covered surface area of each sample was approximately 10^2^ cm^2^ (10 cm × 10 cm), except for elevator buttons. The swabbing was performed according to EN ISO 18593:2018. Sampling from the same sites was repeated by the same trained professional on 23 May 2022 (sampling phase 2) and on 3 June 2022 (sampling phase 3), before routine cleaning and decontamination, as on 14 May 2022 (sampling 1), this making 360 samples in total (120 per sampling phase).

### 2.3. Application of NPS 360°

The coating was applied once to surfaces of Sepolia Station and Wagon 69M317 on 15 May 2022, in accordance with the manufacturer’s instructions. Sepolia Station and Wagon 69M317 operated a few hours later, with no disruption of transportation services throughout the entire study period.

### 2.4. Laboratory Detection of Microbial Load

Samples were tested for bacteria and fungi at the Department of Biopathology of the Aeginition Hospital (University of Athens Medical School), as described elsewhere [31]. Identification of bacteria and fungi was performed by use of the MicroScan autoSCAN-4 System, Beckman Coulter. Briefly, the swabs were broken and submerged in a microtube containing 2.5 mL of sterilized buffered peptone water as an elution medium. The microtubes containing broken swabs were vortexed for 20 s. One ml of supernatants was then used for further quantitative determination analyses. Elution medium swabs containing bacteria or fungi were cultured on conventional solid media covering a broad spectrum of Gram-positive and Gram-negative bacteria and fungi: blood agar, chocolate agar, MacConkey agar, Sabouraud—dextrose agar, mannitol—salt agar (Chapman), *Clostridioides difficile* agar. For each swab 200 μL of elution medium was streaked on a whole agar plate. After incubation for 24 h or 48 h colonies were counted and calculated on the initial volume of 2.5 mL and culture results were expressed in colony forming units (CFUs)/10^2^ cm^2^. All culture media were provided by Bioprepare Microbiology, Keratea—Attica, Greece, manufactured according to ISO 9001:2015–ISO 13485:2016 with CE Mark.

For the detection of SARS-CoV-2, samples were collected from the designated surfaces in Viral Transport Medium and were forwarded to the Hellenic Pasteur Institute (Athens) in cooler bags in order to maintain their integrity. Total RNA extraction was performed with the MagCore Viral Nucleic Acid Extraction kit 203, as recommended by the manufacturer. Viral RNA was assessed by real-time reverse transcription (RT)-PCR, for the detection of the viral envelope protein (E)-encoding gene and the RNA-dependent RNA polymerase (RdRp) gene [32]. The Virus Transport Medium (GLYE), CE-IVD, was supplied by Bioprepare Microbiology.

### 2.5. Statistical Analysis

Categorical variables were presented as percentages. For continuous variables, the median and range were calculated. Poisson regression was used to compare the cumulative number of bacteria after the intervention (comparing the number of bacteria between sampling phases 1 and 2 and sampling phases 1 and 3). *p*-values of ≤ 0.05 were considered statistically significant.

### 2.6. Ethics

The study was approved by the Board of Directors of the Athens underground metro (No 2052, 18 February 2022). The data were managed in accordance with the national and European laws.

## 3. Results

Table 1, Table 2 and Table 3 show the results of surface investigation in the Athens underground metro for bacteria, fungi, and SARS-CoV-2, respectively.

The highest isolation rates concerned samples collected from seats (15 of 60 samples; 25%) and stairs’ handrails (7 of 36 samples; 19.4%) in the station, followed by samples collected from the wagon’s poles (9 of 48 samples; 18.7%). Overall, the isolation rate in the station was 19.7% (26 of 132 samples) and in the wagon 17.5% (40 of 228 samples).

### 3.1. Sampling Phase 1

Before the application of the coating, 60 (50%) of 120 samples tested positive, as follows: 55 (91.7%) of 60 samples tested positive for bacteria and/or fungi, including 32 samples with more than one isolate and 5 (8.3%) of 60 samples tested positive for SARS-CoV-2. In five sampled surfaces, SARS-CoV-2 and bacteria were concomitantly detected. Table 4 shows the detected bacteria and fungi by sampling site and phase.

Coagulase-negative *Staphylococcus* (CNS) was the prevalent bacterium isolated (36 samples), followed by *Staphylococcus aureus* (11 samples), *Micrococcus* spp. (10 samples), *Corynebacterium* spp. and *Acinetobacter Iwoffii* group (8 samples each), and *Bacillus* spp. (7 samples). *Aspergillus* spp. (mainly *A. niger*) accounted for 9 (75%) of 12 detected fungi. Overall, 40 (66.6%) samples were contaminated with staphylococci. Before the application of the coating, a median of 8.5 CFU/10^2^cm^2^ was found for bacteria (range: 0–200 CFU/10^2^cm^2^, IQR 28).

### 3.2. Sampling Phases 2 and 3

Nine days after the application of the coating (sampling phase 2), 2 (3.3%) of 60 samples tested positive for CNS; no other microorganism was detected at that time (Table 4). Sampling phase 3 yielded positive 4 (6.6%) of 60 samples and CNS accounted for four of five isolates (Table 4). In sampling phases 2 and 3, a median of 0 (range 0–1, IQR 0) and 0 (range 0–1, IQR 0) CFU/10^2^cm^2^ was estimated for bacteria, respectively. The application of the coating was estimated to reduce by 100% (RR = 0.001; 95% CI: 0.000–0.005; *p*-value < 0.001) the bacterial load in phase 2 and 95% (RR = 0.053; 95% CI: 0.034–0.083; *p*-value < 0.001) in phase 3. All samples collected after the application of the coating (sampling phases 2 and 3) tested negative for fungi and SARS-CoV-2 (100% reduction).

## 4. Discussion

Reports from application of self-decontaminating coatings in healthcare settings indicate promising results [7,33], while in vitro studies show very good results with usnic-acid-containing self-decontaminating coatings [34,35,36]. To the best of our knowledge, there are no published data on the effectiveness of an usnic-acid-containing self-decontaminating coating using real-life data.

The aim of the current study was to assess the effectiveness of an usnic-acid-containing self-decontaminating coating in the Athens underground metro. We selected a range of heavily touched surfaces from a station and a wagon and found excellent antimicrobial effectiveness against bacteria, fungi, and SARS-CoV-2. Indeed, the application of the coating resulted in the elimination of microbial contamination on all investigated surfaces.

Before the application of the coating, 91.7% of our surface samples tested positive for bacteria and/or fungi. Our results are in line with the findings in underground metros globally, showing the abundance of Staphylococci but also *Corynebacterium* spp., *Bacillus* spp., and *Acinetobacter* spp. [12]. Similarly, 242 (75.6%) of 320 surface samples from the Guangzhou metro system were contaminated with Staphylococci, including 193 (79.75%) MDR strains of various potential sources [11]. *Staphylococcus* spp. also prevailed in surfaces from subway stations in Oslo, Norway, along with other hand bacteria [8]. Overall, as reported by others [9,10,13], the bacterial environmental burden in the Athens underground metro mostly resembles the composition of a mixture of human skin commensals from diverse passengers. Carbapenem-resistant and mcr-1-mediated colistin-resistant *Enterobacteriae* have been also detected in frequently touched surfaces in the Beijing underground metro and the Guangzhou public transportation system, respectively [37,38], while indications of clinically relevant antibiotic-resistant gene transmission have been found in the Hong Kong underground metro [13]. Notably, before the application of the coating, we found a median of 8.5 CFU/10^2^cm^2^ for bacteria, with values as high as 200 CFU/10^2^cm^2^. At the moment, there are no established limits for assessing the presence of bacteria on surfaces in public areas. Nevertheless, the heavy microbial environmental contamination in underground metros indicates that current cleaning and disinfecting procedures are largely insufficient and that efficient, long-term decontamination strategies are needed.

In our study, before the application of the coating, 8.3% of 60 surface samples tested positive for SARS-CoV-2. Among other factors, the prevalence of SARS-CoV-2 infections in passengers may account for differences across studies, as well as compliance with infection-prevention measures. For instance, higher (40%) positivity rates have been detected in surface samples from subway wagons in Barcelona, which, in several cases, persisted after nocturnal maintenance and cleaning, indicating heavy contamination and gaps in disinfection practices [17]. However, the detection of SARS-CoV-2 RNA by highly sensitive real-time PCR does not imply infectiousness.

As expected, in our study, the highest isolation rates were found in station seats, stairs’ handrails, and wagon poles, which are attributed to the continuous and firm contact of multiple hands with these objects throughout the day [12]. In the Guangzhou metro study, escalators and handrails had the higher contamination rate [11]. It is suggested that the higher porosity of rubber handrails may amplify contact surface and harbor nutrients that facilitate bacterial growth [12]. Metallic objects (e.g., station seats and wagon poles) may repel microbial adherence, yet they provide the ideal substrate for fast microbe exchange [12].

Clear strengths of our study include the estimation of the antimicrobial effectiveness of the coating using real-life data and a large number of samples from highly touched surfaces. A limitation is that samples were not collected beyond 20 days after the application of the coating. Another potential limitation is that the subsequent collection of two samples from each surface could limit the microbial load on the tested surface.

## 5. Conclusions

Our study offers an insight on contamination burden in indoor mass transportation environments and particularly on their role in microbe–host interactions. Our study also addresses the need for evidence-based evaluation of cleaning and decontamination procedures in public settings. We showed that the application of an usnic-acid-containing self-decontaminating coating eliminated bacterial, fungal, and SARS-CoV-2 load in highly touched surfaces of the Athens underground metro. Our findings support the wide use of an usnic-acid-containing self-decontaminating coating in underground metros and may also guide infection-control strategies for other heavily occupied public indoor environments.

## Figures and Tables

**Table 1:** Samples testing positive for bacteria by sampling site and phase, Athens metro.

Sampling Sites	Sampling 1 *	Sampling 2 *	Sampling 3 *
**Underground Station**			
stairs handrails †	5/6	0/6	1/6
elevators’ buttons	2/4	0/4	0/4
tickets counter	2/2	0/2	0/2
seats	10/10	1/10	1/10
**Wagon**			
doors (inner surface)	5/5	0/5	0/5
poles	8/8	0/8	0/8
handles	13/15	0/15	2/15
seats	9/10	1/10	0/10

* sampling 1: 1 h before the application of the coating; sampling 2: 9 days after the application of the coating; sampling 3: 20 days after the application of the coating; † includes 2 samples from escalators’ handrails.

**Table 2:** Samples testing positive for fungi by sampling site and phase, Athens metro.

Sampling Sites	Sampling 1 *	Sampling 2 *	Sampling 3 *
**Underground Station**			
stairs handrails †	0/6	0/6	0/6
elevators’ buttons	0/4	0/4	0/4
tickets counter	0/2	0/2	0/2
seats	2/10	0/10	0/10
**Wagon**			
doors (inner surface)	3/5	0/5	0/5
poles	0/8	0/8	0/8
handles	4/15	0/15	0/15
seats	2/10	0/10	0/10

* sampling 1: 1 h before the application of the coating; sampling 2: 9 days after the application of the coating; sampling 3: 20 days after the application of the coating; † includes 2 samples from escalators’ handrails.

**Table 3:** Samples testing positive for SARS-CoV-2 by sampling site and phase, Athens metro.

Sampling Sites	Sampling 1 *	Sampling 2 *	Sampling 3 *
**Underground Station**			
stairs’ handrails †	1/6	0/6	0/6
elevators’ buttons	0/4	0/4	0/4
tickets’ counter	0/2	0/2	0/2
seats	3/10	0/10	0/10
**Wagon**			
doors (inner surface)	0/5	0/5	0/5
poles	1/8	0/8	0/8
handles	0/15	0/15	0/15
seats	0/10	0/10	0/10

SARS-CoV-2: severe acute respiratory syndrome coronavirus 2; * sampling 1: 1 h before the application of the coating; sampling 2: 9 days after the application of the coating; sampling 3: 20 days after the application of the coating; † includes 2 samples from escalators’ handrails.

**Table 4:** Isolated bacteria and fungi by sampling site and time, Athens underground metro.

No	Sampling Site	Sampling 1 * (CFU/10^2^ cm^2^)	Sampling 2 * (CFU/10^2^ cm^2^)	Sampling 3 * (CFU/10^2^ cm^2^)
1	stair handrail	*Bacillus* spp (80)	no growth	CNS (1)
2	stair handrail	no growth	no growth	no growth
3	stair handrail	Gram(+) bacterium (40), *Escherichia coli* (6)	no growth	no growth
4	stair handrail	CNS (6), *Staphylococcus aureus* (1)	no growth	no growth
5	stair handrail	*Acinetobacter lwoffii* group (55)	no growth	no growth
6	stair handrail	*Acinetobacter lwoffii* group (34)	no growth	no growth
7	elevator button	no growth	no growth	no growth
8	elevator button	CNS (1)	no growth	no growth
9	elevator button	no growth	no growth	no growth
10	elevator button	*Corynebacterium* spp (40)	no growth	no growth
11	seat	*Corynebacterium* spp. (10), CNS (3), *Staphylococcus aureus* (1)	no growth	no growth
12	seat	CNS (3)	CNS (1)	no growth
13	seat	CNS (6)	no growth	no growth
14	seat	*Acinetobacter baumanii* (80)	no growth	no growth
15	seat	CNS (25), *Micrococcus* spp. (2), *Staphylococcus aureus* (1) saprophytic hyphae (1)	no growth	no growth
16	seat	CNS (90), *Aspergillus fumigatus* (1)	no growth	no growth
17	seat	*Micrococcus* spp. (12), CNS (3)	no growth	no growth
18	seat	*Micrococcus* spp. (5), CNS (2)	no growth	no growth
19	seat	*Micrococcus* spp. (2), *Staphylococcus aureus* (6), *Escherichia coli* (8)	no growth	CNS (1), *Micrococcus* sp. (1)
20	seat	CNS (2)	no growth	no growth
21	tickets’ counter	*Acinetobacter lwoffii* group (120), *Staphylococcus aureus* (2)	no growth	no growth
22	tickets’ counter	CNS (24)	no growth	no growth
23	pole	*Acinetobacter lwoffii* group (160)	no growth	no growth
24	pole	*Acinetobacter lwoffii* group (24), CNS (1)	no growth	no growth
25	pole	CNS (6), *Klebsiella* spp. (8), *Bacillus* spp. (10)	no growth	no growth
26	pole	*Bacillus* spp. (4), CNS (2)	no growth	no growth
27	pole	CNS (2), *Micrococcus* spp. (2), *Corynebacterium* spp. (2)	no growth	no growth
28	pole	*Acinetobacter lwoffii* group (170)	no growth	no growth
29	pole	*Staphylococcus aureus* (2)	no growth	no growth
30	pole	*Staphylococcus aureus* (9), CNS (2)	no growth	no growth
31	door	CNS (6)	no growth	no growth
32	door	CNS (7), *Aspergillus niger* (1)	no growth	no growth
33	door	CNS (2), *Corynebacterium* spp. (1)	no growth	no growth
34	door	CNS (6), *Aspergillus niger* (2), *Aspergillus flavus* (1)	no growth	no growth
35	door	CNS (94), *Escherichia coli* (3), *Staphylococcus aureus* (36), *Aspergillus niger* (1)	no growth	no growth
36	handle	*Pantoea agglomerans* (7), CNS (1)	no growth	no growth
37	handle	CNS (6), *Corynebacterium* spp. (5)	no growth	no growth
38	handle	CNS (2), *Micrococcus* spp. (3)	no growth	no growth
39	handle	*Aspergillus fumigatus* (1)	no growth	no growth
40	handle	*Acinetobacter lwoffii* group (6), *Bacillus* spp. (6)	no growth	no growth
41	handle	*Escherichia coli* (2), *Bacillus* spp. (1)	no growth	no growth
42	handle	*Acinetobacter lwoffii* group (14)	no growth	no growth
43	handle	CNS (1)	no growth	CNS (1)
44	handle	*Micrococcus* spp. (2), *Aspergillus niger* (2)	no growth	no growth
45	handle	CNS (2), *Corynebacterium* spp. (1), saprophytic hyphae (1)	no growth	CNS (1)
46	handle	*Staphylococcus aureus* (3), CNS (6)	no growth	no growth
47	handle	CNS (2)	no growth	no growth
48	handle	no growth	no growth	no growth
49	handle	CNS (13)	no growth	no growth
50	handle	*Staphylococcus aureus* (4), *Micrococcus* spp. (3), *Aspergillus niger* (2)	no growth	no growth
51	seat	*Staphylococcus aureus* (2), CNS (5)	no growth	no growth
52	seat	*Klebsiella* spp. (200) environmental blastomycetes (8)	no growth	no growth
53	seat	*Bacillus* spp. (30)	no growth	no growth
54	seat	CNS (6)	no growth	no growth
55	seat	CNS (25), *Corynebacterium* spp. (10)	no growth	no growth
56	seat	*Escherichia coli* (12), *Micrococcus* spp. (15), CNS (3), *Aspergillus niger* (1) *Bacillus* spp. (2)	no growth	no growth
57	seat	CNS (1), *Micrococcus* spp. (1), *Corynebacterium* spp. (1)	no growth	no growth
58	seat	CNS (90)	no growth	no growth
59	seat	CNS (10)	CNS (1)	no growth
60	seat	no growth	no growth	no growth

CFU: colony-forming unit; CNS: coagulase-negative *Staphylococcus*; * sampling 1: 1 h before the application of the product; sampling 2: 9 days after the application of the product; sampling 3: 20 daysafter the application of the product.

## Data Availability

Data are available upon request.
